# Plant nuclear envelope as a hub connecting genome organization with regulation of gene expression

**DOI:** 10.1080/19491034.2023.2178201

**Published:** 2023-02-16

**Authors:** Yu Tang

**Affiliations:** Peking University Institute of Advanced Agricultural Sciences, Shandong Laboratory of Advanced Agricultural Sciences at Weifang, Weifang, Shandong, China

**Keywords:** Nuclear envelope, nuclear pore complex, nuclear lamina, nuclear membrane, plants, gene expression, genome organization

## Abstract

Eukaryotic cells organize their genome within the nucleus with a double-layered membrane structure termed the nuclear envelope (NE) as the physical barrier. The NE not only shields the nuclear genome but also spatially separates transcription from translation. Proteins of the NE including nucleoskeleton proteins, inner nuclear membrane proteins, and nuclear pore complexes have been implicated in interacting with underlying genome and chromatin regulators to establish a higher-order chromatin architecture. Here, I summarize recent advances in the knowledge of NE proteins that are involved in chromatin organization, gene regulation, and coordination of transcription and mRNA export. These studies support an emerging view of plant NE as a central hub that contributes to chromatin organization and gene expression in response to various cellular and environmental cues.

## Introduction

Nuclear envelope (NE) encloses genomic DNA and separates the nucleoplasm from the cytosol. This two-layered physical barrier is comprised of the outer nuclear membrane and inner nuclear membrane (INM), which are compositionally distinct with regard to their membrane proteome [1]. NEs are perforated by ring-shaped pores, known as nuclear pore complexes (NPCs), which serve as selective transport gates for soluble macromolecules. Beneath the INMs are the intermediate filament lamins, which form a polymeric meshwork and contribute structural support for the nucleus [2].

NE acts as both a barrier and an anchor for chromosomes. As illustrated in both animals and plants, the nuclear periphery is generally considered as a transcriptionally repressive environment due to the presence of heterochromatin and enriched with repressive epigenetic marks [3,4]. Nuclear lamina (NL) comes in close and direct contact with the underlying heterochromatin and has been implicated in numerous chromatin-associated processes [5–7]. Moreover, the NL is responsible for the proper retention and localization of INM proteins (e.g., human Sad1–UNC-84 proteins and Arabidopsis INM protein Plant Nuclear Envelope Transmembrane 2) [8,9]. Those INM-resident components in Opisthokonts, on the other hand, have been well studied to engage in spatial genome organization, DNA replication and repair, and transcriptional regulation through an intricate interplay with chromatin and chromatin regulatory factors [10].

Even though tightly packed heterochromatin preferentially locates to the nuclear periphery, the less condensed euchromatin regions may also position around the nuclear pore [11,12]. Except for its core function of transport, the NPC has been shown to be associated with the underlying chromatin, as well as multiple chromatin remodeling and transcriptional complexes, contributing to diverse gene regulatory processes [13]. Recent evidence in animals and yeast suggests that the NPCs may be more dynamic and heterogeneous in both composition and conformation than previously expected, suggesting structural and functional plasticity for cargo transport and gene regulation [14–18]. Several recent reports emphasize that the nuclear pore is also intimately connected with the nucleoskeleton and is critical for the proper maintenance of lamin structure, NPC distribution and homeostasis, chromatin architecture, and gene regulation in Opisthokonts [19–21]. However, such mechanistic information on plant NPC is still rare. Recently, the physical and functional connection between nuclear pores and nucleoskeletons in Arabidopsis has been established by the identification and characterization of a novel plant nucleoporin (see below) [22].

In humans, a variety of physiological processes and pathologies, including numerous genetic disorders and cancers, are intimately linked to mutations of the NE constituents and mostly determined by its functional connection with genome organization [23]. Using Arabidopsis as the model organism, research on the plant NE supports its critical roles in genome activities and has expanded its function to regulating cellular responses to diverse developmental and environmental signals [24,25]. In this review, I aim to outline the current knowledge on the role of plant NE-resident components and to illustrate their connections to genome organization, transcriptional regulation, and epigenetic maintenance.

## The nuclear lamina is an organizational platform for genome architecture

**Figure 1.**
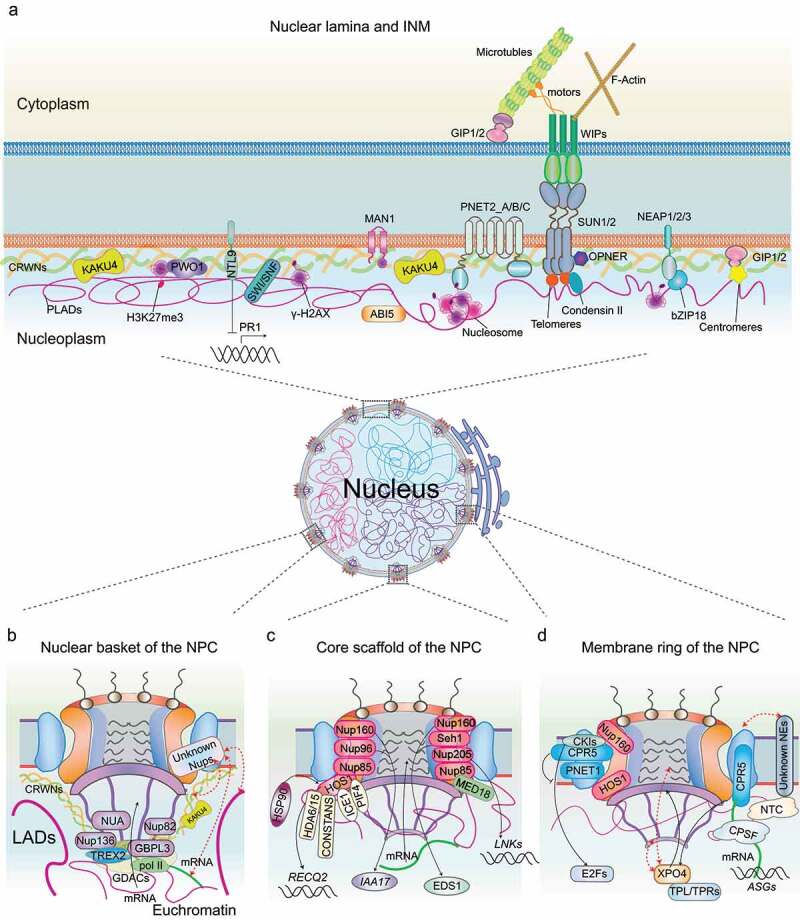
Summary of plant key regulators at the nuclear envelope involved in genome organization and regulation of gene expression. (a) The plant nuclear periphery mostly features a high concentration of heterochromatin regions with repressive epigenetic marks, termed plant lamina-associated domains (PLADs). Plant nucleoskeleton four CRWN proteins are responsible for chromatin tethering as well as association with chromatin regulators (e.g. H3K27me3, PRC2, SWI/SNF, NTL9, and ABI5). KAKU4 is another plant potential nucleoskeleton component. Plant INM resides some conserved proteins (e.g. MAN1, PNET2s, and SAD/UNC homology (SUNs)), as well as plant-specific components (e.g. NEAPs, GIPs, and OPENER), and is involved in multiple aspects of genome activity regulation. The nuclear basket (b), the core scaffold (c), and the membrane ring (d) of the NPC encompass distinct nucleoporins that are associated with underlying chromatin and/or chromatin-associated complexes. The speculative connections at the NE are highlighted in red dashed lines (b, d), including nucleoporins with the underlying nucleoskeleton, chromatin, and RNA; nuclear membrane constituents with the NPC.

NL is composed of lamin proteins and other proteins associated with the nucleoskeleton and forms a dense filamentous protein network underneath the INM [26] (Figure 1a). Comparative and systematic analyses of lamins among different eukaryotes have shown that lamin or lamin-like proteins are retained across highly divergent species from metazoans to plants but are absent in yeast cells [27,28]. Lamins in animals and nuclear matrix constituent proteins (NMCPs) in plants are central nucleoskeleton components that display remarkable evolutionary plasticity in protein size and sequence but share a comparable tripartite structure with a highly conserved central coiled-coil rod domain flanked by less conserved short head and long tail domains [24,29]. The NMCP-related Arabidopsis proteins are known as CROWDED NUCLEI (CRWNs), which can be divided into two subfamilies: clade 1 NMCPs (CRWN1, CRWN2, and CRWN3) and clade 2 NMCP (CRWN4) based on their evolutionary distance [30,31]. These plant functional lamin-like counterpart NMCPs/CRWNs physically interact with each other and organize into the NL by forming a filamentous network structure [32,33]. Four CRWN proteins are predominantly located at nuclear periphery, while CRWN2 and CRWN3 are also partially located in the nuclear interior, indicating their potential divergent roles in the nucleus [33–36].

### Nuclear lamina is directly involved in chromatin tethering

As in animals, the plant nuclear peripheral zone is also in a repressed chromatin environment, enriched with considerable transposable elements, and silenced protein-coding genes and heterochromatic marks [4]. Arabidopsis CRWN1 and CRWN4 play important roles in maintaining nuclear architecture, and both single mutants show reduced nuclear size, altered nuclear morphology, and disordered spatial heterochromatin organization [30]. By leveraging high-throughput chromosome conformation capture (Hi-C) analysis and *in situ* hybridization assays, Hu et al. provided clear evidence that CRWN1 and CRWN4 are responsible for chromatin tethering at nuclear periphery, with the loss of either CRWN1 or CRWN4 resulting in altered chromatin position patterns and attenuated chromatin compartmentalization [37]. Moreover, like metazoan lamin proteins, CRWN1 directly interacts with non-accessible chromatin regions, which are characterized by low-expression genes and repressive chromatin marks such as H3K27me3. Thus, these regions are termed plant lamina-associated domains (PLADs) [37].

However, the nuclear periphery is not always a transcriptionally inactive region. Some highly expressed genes are also found at the nuclear periphery [4]. Certain external environmental cues influence chromatin positioning, accompanied by enhanced gene expression at nuclear periphery. For example, upon light stimuli, light-inducible chlorophyll a/b-binding protein (CAB) gene loci were found to rapidly relocate from the nucleoplasm to the nuclear periphery, which is more likely an independent regulatory step before full transcriptional activation [38]. Furthermore, CRWN1 has been recently discovered to play a role in activation of copper-associated genes (CAs) [33]. Under excess copper conditions, CRWN1 at the nuclear periphery directly interacted with CAs, which are tandemly localized on chromosomes in clustered loci. Thereby, this CRWN1-associated gene repositioning promotes the expression of CAs to regulate copper tolerance in plants [33].

### Nuclear lamina participates in regulating diverse genome activities

In line with the role of CRWNs in chromatin tethering, transcriptome analyses using higher-order *crwn* mutants show that CRWNs are critical for regulation of gene expression. Among the four CRWNs, CRWN1 is the prominent component of the nucleoskeleton-chromatin network and acts as an important regulator of PAMP-triggered immunity (PTI) responses and jasmonic acid signaling [37,39]. Loss of *crwn1* caused compromised expression of PTI-responsive genes and upregulation of JA-responsive genes, subsequently resulting in susceptibility to the non-virulent *P. syringae pv. tomato DC3000 hrcC−* (*Pst hrcC−*) pathogen and increased endogenous JA levels [39]. Single mutants of *CRWN* paralog display a high degree of overlap in the misregulated differentially expressed genes (DEGs) and different combinations of *crwn* mutants, especially *crwn1 crwn2* and *crwn1 crwn4*, show comparable numbers of up-regulated and down-regulated DEGs that were evenly distributed on five chromosome arms, suggesting that CRWNs may undergo extensive interactions with chromatin and play a general role in whole-genome transcriptomic regulation [33,40,41]. Interestingly, the upregulated genes were significantly enriched in multiple biotic and abiotic stimuli, including Salicylic Acid (SA) mediated defense signaling and abiotic stress responses [40–43]. Similarly, a study of NMCP in the liverwort showed that loss of NMCP resulted in activated biotic and abiotic responses, revealing an essential and conserved role of the nucleoskeleton in regulating stress responses across the plant kingdom [44]. Mechanistic details of how the nucleoskeleton is involved in stress response regulation are still rare. During Arabidopsis seed germination, CRWN1 and CRWN2 are required for the increase in the nuclear size, accompanied by the reduced chromatin compaction. This response is dependent on seed maturation regulator ABSCISIC ACID-INSENSITIVE 3 (ABI3), suggesting a functional link between ABA signaling and the nucleoskeleton [45]. Another piece of evidence is the association between CRWN3 and a basic leucine zipper (bZIP) transcription factor ABA INSENSITIVE 5 (ABI5) in ABA signaling [46]. Interestingly, CRWN3 has been proposed to participate in seed germination by mediating the degradation of ABI5 protein in the nuclear bodies [46]. Besides ABA, the absence of CRWNs displayed attenuated H3K27me3 modification levels near genes that encode transcription factors mediating SA biosynthesis, suggesting a tight connection of CRWN1 and H3K27me3 mark in repressing chromatin at the nuclear periphery [40,42]. Alterations of such epigenetic modifications may cause DNA oxidative damage [47]. Consistently, the genome of *crwn* mutants suffers from more severe DNA damage upon oxidative stress, indicating that CRWNs contribute to DNA repair upon spontaneous damage, possibly via the association with these epigenetic marks [48]. In line with this observation, in plants, the DNA damage and repair marker γ-H2AX form foci at DSB sites, which are frequently localized to the nuclear periphery. Loss of CRWN1/4 compromised the peripheral localization of those foci after γ-irradiation, suggesting that NL contributes to the protection of genome stability [49].

### Nuclear lamina associates with a diverse arrays of chromatin regulators

In addition to their role in chromatin tethering, CRWNs also directly interact with chromatin regulatory factors in Arabidopsis. One outstanding example is PWO1, a component of the polycomb repressive complex 2 (PRC2), which trimethylates lysine 27 on histone H3 (H3K27me3) [41,50]. PWO1 predominantly forms subnuclear speckles and physically interacts with CRWN1 at the nuclear periphery, adding another proof for the connection between H3K27me3-marked chromatin and the NL in plants. Loss of PWO1 or CRWN1 resulted in similar effects in Arabidopsis, such as decreased nuclear size and activated stress responses, and a comparable set of misregulated genes, indicating that CRWN1 and PWO1 function together in the maintenance of nuclear architecture and chromatin regulation [41]. Consistent with this, lamin-like protein OsNMCP1 physically interacts with SWITCH/SUCROSE NONFERMENTING (SWI/SNF) chromatin remodeling complex to modulate chromatin accessibility and organization in rice root growth and drought resistance [51]. In addition, a membrane-bound NAC transcription factor, NAC WITH TRANSMEMBRANE MOTIF1-LIKE9 (NTL9) has been implicated in salicylate-mediated immune responses, showing association with CRWN1 [43]. The interaction between CRWN1 and NTL9 enhanced the promoter-binding activity of NTL9, which represses the transcriptional output of PATHOGENESIS-RELATED1 (PR1), a defense-inducible marker gene, suggesting a positive role of CRWN1 in gene silencing [43].

Besides CRWNs, the plant genome evolved a unique putative nucleoskeleton protein KAKU4 [52]. Loss of KAKU4 in Arabidopsis resulted in aberrant nuclear morphology, displaying spherical nuclei, similar to those in *crwn1* or *crwn4* mutants. In addition, the ectopic expression of Arabidopsis KAKU4 induces the deformation of nuclear membrane and this effect could be enhanced by overaccumulation of CRWN1, which phenocopied the nuclear deformation caused by overexpression of nucleoskeleton proteins in maize [52,53]. These studies together indicate KAKU4 and CRWN1 coordinately maintain nuclear morphology and overall nuclear architecture of plants. The N-terminal conserved domains of KAKU4 are responsible for its physical interactions with CRWN1/4 for nuclear shape organization at nuclear periphery (bioRxiv preprint, also see below nuclear basket part) [54]. A recent study taking advantage of proximity labeling proteomics revealed that many nucleosome assembly, histone binding, and DNA binding-related proteins are part of KAKU4 proxitome (by proximity-labeling proteomics), suggesting KAKU4 at the NL is intimately associated with nucleosome-related chromatin [9]. More intriguingly, KAKU4 protein bears a Arg- and Gly-rich region, which has a potential RNA binding affinity in the nucleus [52]. Together, these studies in plants have revealed multi-layered regulation of NL, from maintaining nuclear architecture to regulating chromatin organization and transcription.

## Inner nuclear membrane contains diverse regulators of chromatin organization and gene expression regulation

Compared to compartments of the ONM and its continuous endoplasmic reticulum (ER), the INM hosts a unique collection of membrane proteins that maintain close contact with both NL and chromatin [10]. Recent advances have expanded the list of INM candidates to several hundred, and at least 35 integral membrane proteins have been verified to localize and function at the INM in mammalian cells [10,55]. These Opisthokont INM components serve a wide range of nuclear and cellular functions, including but not limited to chromatin organization, gene expression regulation, maintenance of genome stability, mechanosensation and mechanotransduction, and signaling transduction into nucleus [10]. Due to plant genomes do not contain homologs of most known opisthokont NE proteins, the number of characterized INM proteins in plants is far less than humans and yeast [9,56]. Recently, by combining subtractive proteomics and proximity-labeling technology, Tang et al. successfully identified a series of plant NE proteins, which also include some uncharacterized INM components [56]. In this part, I illustrate these examples of well-known and newly discovered plant INM constituents to describe their functions in genome-related processes (Figure 1a).

### MAN1 protein is a conserved INM component in plants

MAN1 is a conserved INM component and exists in many species, including animals, yeast, and plants [57]. Unlike animal and yeast MAN1, plant homologs of MAN1 lost its feature domain, LAP2-emerin-Man1 (LEM) domain in its N terminus, which renders interaction with the chromatin-binding protein barrier-to-autointegration factor (BAF) [57]. Plant MAN1 retained two transmembrane helixes and a conserved Man1-Src1p carboxy-terminal domain, which may still confer its DNA-binding function at nuclear membrane in plants [58]. Indeed, AtMAN1 is predominantly located at the nuclear membrane [9]. Moreover, proximity labeling proteomics of AtMAN1 revealed that AtMAN1 protein significantly probed nucleoskeleton proteins CRWN1/4, indicating AtMAN1 is located at INM near the NL. Other prominently enriched interactors of AtMAN1 include another INM protein PNET2_A (see below), as well as a couple of putative chromatin regulators, such as histone modifiers, transcription factors, and certain DNA binding proteins [9]. Both localization and potential interactors of AtMAN1 indicate that MAN1 resides at INM and may contribute to chromatin tethering in the plant kingdom. However, loss of AtMAN1 does not show observable developmental phenotypes under normal conditions [9]. Future studies will further elucidate its potential function in chromatin binding and regulation.

### PNET2 family is the critical player of genome organization and activity in plants

Plant PNET2 protein family was identified by both subtractive proteomics for the general NE proteome profiling and proximity-labeling proteomics using AtMAN1 as bait [9,56]. PNET2 proteins are relatively conserved INM components with multi-transmembrane helices that exist in both animals and plants but bear significant sequence divergence. PNET2 has three paralogs in Arabidopsis, including PNET2_A, PNET2_B, and PNET2_C. These PNET2 are preferentially enriched at NE and bind to nucleoskeleton by physical interaction between their N-terminus and CRWN1. Interestingly, transient co-overexpression of soluble N-terminus of PNET2 and CRWN1 induces the formation of subnuclear condensates, which may contribute to the establishment of chromatin-rich compartment at NL. On the other hand, the C-terminus of PNET2 induces formation of homomeric complexes and also promotes liquid–liquid phase separation in nucleus. Interestingly, the PNET2 C-terminus can be independently recruited to the nuclear periphery by KAKU4 protein. These data together indicated that PNET2 is intimately associated with plant nucleoskeleton components and is part of NL in plants [9].

Proximity labeling proteomics revealed that PNET2 proteins at NL are preferentially associated with core histone components, and PNET2 was able to pull down native histone H2A protein in vivo, demonstrating the close chromatin association of PNET2 family in plants. The *pnet2* mutant exhibits globally reprogramed chromatin architecture including enhanced local intra-chromosomal interaction, attenuated distant intra- and inter-chromosomal interactions, and altered interactions between both euchromatic and heterochromatic chromocenters on all chromosomes. CRISPR Cas9-based mutations of all three *PNET2* genes in Arabidopsis can cause severe growth restriction and finally lead to seedling lethality, indicating PNET2 is important for plant survival. Loss of PNET2 induces widespread transcriptional changes, which disrupted the steady-state gene expression levels of growth-versus-stress. Together, these analyses revealed that PNET2s are critical for engaging in chromatin organization and maintaining proper genome activity in plants [9,59].

### Plant SUN proteins are associated with both telomeres and centromeres

INM-localized SUN proteins undergo trimerization, which in turn associates with Klarsich Anc Syne Homology (KASH) proteins at the ONM to form the linker of nucleoskeleton and cytoskeleton (LINC) complex [60,61]. Binding of cytoskeletal components by KASH proteins and the association between SUN proteins with nuclear lamins and chromatin allow the LINC complex to act as a bridge connecting the cytoskeleton and nucleoskeleton [62]. SUN proteins share significant homology among diverse species and feature with the conserved SUN (Sad1_UNC) domain located in the perinuclear space of the NE. Besides the SUN domain, most SUN proteins also bear one or multiple transmembrane domains and coiled-coil domains [63,64]. SUN proteins can be grouped into two distinct clusters, including the classical C-terminal SUN domain containing proteins (C-ter SUNs) and the atypical middle SUN domain containing proteins (mid-SUNs) [65]. Arabidopsis SUN proteins contain two C-ter SUNs, AtSUN1 and AtSUN2, and three mid-SUNs, AtSUN3, AtSUN4, and AtSUN5. AtSUN1/2 are relatively conserved INM components and are closely associated with nucleoskeleton CRWN proteins, while mid-SUNs are both INM and ER localized in Arabidopsis [36,63,64,66]. Although SUN proteins are relatively conserved between animals and plants, the sequence of plant KASH proteins is different from their animal counterparts [67]. In Arabidopsis, WPP domain-interacting proteins (WIPs), are functional KASH and engage another ONM protein family, WPP domain-interacting tail-anchored proteins (WITs) to form the SUN-WIP-WIT LINC complex [68]. The prevalent function of the plant LINC complex is to maintain nuclear shape, size, and movement in different types of cells, which widely affects both plant vegetative and reproductive growth [25,69,70]. Recently, a pioneering study by Gumber et al. has reported the comprehensive constituents of the LINC complex and the physical interactions between SUN and KASH proteins in maize [71]. Interestingly, the LINC complex in maize displays developmental co-expression clustering in a tissue-specific manner, which is not found in Arabidopsis [56]. These results together suggest the functional conservation and divergence of the LINC family in a model grass species [71].

One highly conserved role of SUN proteins in eukaryotes is responsible for tethering and repositioning of telomeres within the NE during meiosis prophase I [72–76]. Loss of SUN proteins in Arabidopsis, rice, and maize causes severe meiotic defects in telomere clustering and crossover formation, resulting in dramatically reduced fertility [72,75–77]. Interestingly, a meiotic-specific structure named SUN Belt has been discovered in maize. The SUN Belt includes telomere bouquet and is involved in meiotic telomere dynamics at zygotene stage [76]. These findings collectively demonstrate the functional conservation of SUN proteins in meiotic progression. In Dictyostelium, SUN-1 provides an intimate link between chromatin and centromere and maintains genome stability by a direct interaction between SUN proteins and chromatin [78]. In Arabidopsis, mutants of *SUN* genes displayed alleviated transcriptional repression of both centromeric and pericentromeric repeats, suggesting that the LINC complex potentially contributes to proper heterochromatin organization and transcriptional gene silencing [79]. Yuki et al. recently reported that LINC complex plays an essential role in the maintenance of the centromere distribution from late anaphase to telophase due to physical interactions between SUN proteins, centromeres, and Condensin II complex [80]. Consistently, mutants of *SUN* or *KASH* genes display altered centromere distribution compared with *crwn* mutants. In contrast, *crwn1 crwn4* contains fewer numbers of centromeres and their movements are more dynamic. Correspondingly, CRWNs are able to interact with centromere components to directly restrain centromere positioning and in turn maintain genome integrity after the entry into interphase [80].

An essential single copy protein OPENER in Arabidopsis was identified as a partner of SUN proteins [81]. Loss of OPENER in Arabidopsis affects cell proliferation and cell cycle progression in both reproductive and vegetative tissues. Interestingly, OPENER is a dual NE and mitochondrial localized protein, and its NE localization is dependent on its association with AtSUN1/SUN2 [81]. This finding emphasizes the influence of SUN proteins and its associated partners on cell cycle progression within the INM.

### The plant NEAP family has the potential to associate with chromatin

More support for a connection between the nuclear periphery and chromatin organization is from the study of Nuclear Envelope-Associated Protein (NEAP) [82], which was first identified via bioinformatic screening. The plant-specific NEAP family is conserved in both angiosperms and gymnosperms, including extensive N-terminal coiled-coil domains, followed by a canonical nuclear localization signal and a C-terminal transmembrane helix. In Arabidopsis, three functional NEAP proteins are predominantly localized at the nuclear periphery and form homomeric or heteromeric complexes [82]. NEAPs preferentially interact with AtSUN1 and AtSUN2 at the nuclear membrane, suggesting that plant NEAPs may be related to nucleocytoskeletal bridging complexes. In addition, AtNEAP1 physically interacts with a putative DNA-binding leucine zipper transcription factor bZIP18, implicating its connection to chromatin at the INM. Functional analyses showed that *neap* mutant displays increased nuclear size and abnormal organization of chromocenter and heterochromatin. Moreover, loss of both *AtNEAP1* and *AtNEAP3* results in restricted primary root growth [82]. Together, these data suggest that the NEAP family is an important plant nuclear membrane component that is linked to both LINC complexes and chromatin.

### Microtubules and γ-tubulin complex protein complex protein 3-interacting proteins (GIPs) are involved in genome stability

The γ-TuC protein 3 (GCP3)-interacting proteins GIP1 and GIP2 were initially identified as regulators involved in proper microtubular component localization for mitotic spindle assembly, as well as for nuclear shape and architecture [83]. In addition, GIPs reside on both sides of the nuclear membrane and recruit centromeric related proteins to maintain centromeric architecture throughout the cell cycle [84]. The *gip1 gip2*-mutant display enhanced resistance to hyperosmotic stress partially contributed by mechanical shielding through nuclear shrinking and stiffening [85]. GIP proteins have also been implicated in the maintenance of genome stability in Arabidopsis, where GIP proteins are predominantly colocalized with the homologous recombination (HR) protein RAD51 at the nuclear periphery [86]. Nuclei in the *gip1 gip2* mutant exhibit abundant and heterogeneous γ-H2AX foci, suggesting constitutive DNA damage and chromosome instability.

## The NPC bridges nuclear transport and gene regulation

As the largest protein assembly, the NPC forms a central channel and serves as a selective transport gate for soluble macromolecules. Each NPC consists of ~1,000 protein subunits with multiple copies of ~40 different proteins (nucleoporins) [87]. Structurally, the NPC is composed of four principal interconnected NPC modules, including the core scaffold, the transmembrane ring, the selective barrier, and the nuclear basket (NB) [88]. The NPC assembly initiates with chromatin-bound core scaffold, which recruits other principal subcomplexes such as the transmembrane ring, the NB, and the channel Nups, to set up the transport barrier [13]. Once assembled, these subcomplexes are associated with the NPC core during interphase and remain seemingly stable and unexchangeable in post-mitotic cells. However, accumulating evidence suggests that those NPC modules are very flexible in conformation in different cellular environments to maintain dynamic communication between the nucleus and cytoplasm [14–17,24].

Although the key role of the NPC is to regulate nucleocytoplasmic transport, a growing body of evidence suggests its involvement in many other nuclear processes, including regulation of gene expression and chromatin architecture [13,89]. The nuclear pore region is enriched with transcriptionally active genes and euchromatin relative to other nuclear peripheral regions [11,90]. Based on these observations, researchers proposed the gene gating hypothesis, in which transcriptionally active genes are tethered next to the NPC in order to facilitate transcription and subsequent rapid nuclear export of transcripts [91]. This hypothesis has been supported by substantial evidence in many species. For example, in yeast, inducible genes such as *GAL, INO1*, and *HXK1* are targeted to the NPC for activation [92–94]. Similarly, in Arabidopsis, the luciferase reporter gene was activated by mimetic fusion to a nucleoporin [95]. However, recent findings in both animals and yeast have shown that the NPC plays a role in both active and repressive activities, with certain NPC components or subcomplexes preferentially affecting gene activation or silencing [13]. Interestingly, the nuclear pore exhibits high heterogeneity at distinct nuclear subdomains even in a single cell, which might be required for organization of chromatin compartmentalization and transport of specific cargos [96]. Taken together, the NPC is clearly a dynamic assembly, with both highly flexible composition and conformation, that accommodates its roles in cargo transport, genome activities and beyond.

### The nuclear basket makes an intimate connection with both chromatin and the nucleoskeleton

NB is assembled by eight flexible protein filaments that emanate from the NPC core and join in a distal ring [88]. The structure of NB displays high flexibility and plasticity, most likely allowing simultaneous associations with chromatin and diverse macromolecular complexes [97,98] (Figure 1b). NB nucleoporins are less conserved between animals and plants than proteins of the NPC core, and only the basket scaffold component TPR is evolutionarily conserved across eukaryotes.

In Arabidopsis, NUA, the functional homolog of TPR, is involved in a variety of biological processes, including reproductive and vegetative development, flowering regulation, and auxin and ABA signaling [99–101]. Compromised NUA led to the retention of mRNA transcripts within the nucleus, suggesting that NUA is also responsible for mRNA export in plants [99,100]. The functional homolog of NUP153 is thought to be AtNUP136, an FG-repeat-containing nucleoporin, which is also associated with mRNA export by directly interacting with TREX-2 complex to regulate both mRNA and miRNAs export [102,103]. This emphasizes a conserved mechanism for mRNA export mediated by the association between the nuclear basket and the TREX-2 complex. In Arabidopsis, NUP82 was identified as a paralog of NUP136 and only exists in plants. So far, there is no direct evidence that NUP136 and NUP82 interact directly with chromatin in plants; however, NUP136 is enriched in pericentromeric regions, as shown by a Restriction Enzyme-Mediated Chromatin Immunoprecipitation (RE-ChIP) and fluorescence *in situ* hybridization (FISH) assay [4,37]. Moreover, NUP136 acts together with NUP82 to positively regulate salicylic acid (SA)-dependent pathogen responses [104]. Plants with loss of NUP136 and NUP82 displayed severe growth defects and impaired to SA-mediated pathogen resistance accompanied with significant downregulation of immune-responsive genes. These findings further underscore the importance of NUP136 and NUP82 in regulating gene expression. However, it remains to be determined whether NUP136 and NUP82 directly bind to chromatin or mediate nucleocytoplasmic transport of specific cargos to regulate immune-responsive gene expression.

A conserved motif is shared by the nb proteins NUP136, NUP82 and nucleoskeleton protein KAKU4. This motif drives their physical interaction with nucleoskeleton CRWN proteins (bioRxiv preprint) [54]. This demonstrates an intriguing molecular mechanism for the direct association between the nucleoskeleton and the nuclear basket in plants, although the functional significance of these associations is still unclear. In support of this notion, NUP136 also plays a role in determining nuclear morphology [105], and the spherical nuclei observed in the *nup136*-mutant partially phenocopy those of nucleoskeleton mutants in Arabidopsis [30,34].

Recently, Tang et al. reported that Guanylate-binding protein (GBP)-like 3 (GBPL3) is predominantly enriched at the NB in Arabidopsis and functionally links the nuclear basket and the nucleoskeleton [22]. GBPL3 physically interacts with components of both NB and the nucleoskeleton in Arabidopsis. Genetic studies indicate that GBPL3 differentially functions with NB and nucleoskeleton. For example, spontaneous loss of GBPL3, NB nucleoporin NUP82 caused stunted growth, early senescence, and a failure to survive and reproduce, indicating that GBPL3 and NUP82 together are functionally essential for plant development. Intriguingly, *GBPL3* displays distinct genetic interactions with the four *CRWN* genes. *gbpl3 crwn1* is seedling-lethal, *gbpl3 crwn2* displays leaf senescence and stunted growth, *gbpl3 crwn3* exhibits leaf senescence and lacks trichomes, *gbpl3 crwn4* shows enhanced SA accumulation and spontaneous chronic lesions on leaves. These results suggest that GBPL3 is associated with different CRWN proteins to selectively regulate plant development and stress responses. Moreover, proximity labeling proteomics showed that GBPL3 promotes the formation of biomolecular condensates, which recruit considerable amount of chromatin remodelers, transcription regulators, and RNA processing machinery components, suggesting a role in gene gating at the NB. In an independent study, Huang et al. reported that immune cues such as SA can drive the formation of GBPL3 defense-activated condensates (GDACs). These nuclear GDACs can recruit the mediator complex of the RNA polymerase II machinery and promote GBPL3 to directly bind promoter regions of defense responsive genes, broadly priming and reprogramming host gene expression for disease resistance [106]. Taken together, these findings support a model that as a novel NPC component, GBPL3 is preferentially enriched at the NB, where it is functionally linked to both the NB and the nucleoskeleton to mediate gene gating by forming biomolecular condensates in plants.

### The core scaffold of the NPC participates in regulating gene expression

The core scaffold of the NPC is structurally and compositionally conserved in many diverse species, including humans, yeast, Xenopus, and algae [98,107–109]. It is highly symmetrical and includes the inner ring complex (IRC) and the outer ring complex (ORC) [88]. The ORC is formed by a conserved Y-shaped NUP107-160 subcomplex in a head-to-tail arrangement. Sandwiched between double ORCs from the cytoplasmic and nucleoplasmic sides, the IRC is anchored at the equatorial plane of the NPC core and is composed of the NUP93 subcomplex (Figure 1c). The cellular environment has a substantial effect on the diameter of the NPC central channel, and the IR is substantially wider in the native state than previously thought [14,15]. This structural plasticity of the NPC may play a central role in efficient transport [16]. The core scaffold is essential for NPC assembly and integrity by intensively interconnecting with other NPC modules and has been implicated in genome regulation via physical interactions with chromatin or chromatin remodelers [13].

Embryo or seedling lethality of the IRC loss-of-function mutants makes it difficult to study the function of IRC nucleoporins in plants using genetics [110]. However, a missense mutation in IRC component NUP205 attenuates defense responses by regulating the expression of a core clock gene family *NIGHT LIGHT-INDUCIBLE AND CLOCKREGULATED* (*LNK*) upon bacterial infection in plants [111]. In contrast to the IRC, most ORC single mutants are able to survive and display pleiotropic phenotypes, suggesting functional diversity of the core scaffold in plants [110]. One of the most documented ORC components, HIGH EXPRESSION OF OSMOTICALLY RESPONSIVE GENES 1 (HOS1) is a homolog of human ELYS. Although it is unclear whether plant HOS1 recruits NUP107-160 complex and mediates NPC assembly as was shown in humans, Arabidopsis HOS1 acts as a chromatin organizer and engages in a multitude of interactions with chromatin regulators. HOS1 directly binds with chromatin or interacts with chromatin remodelers HISTONE DEACETYLASE 6 (HDA6) and HDA15 to epigenetically regulate FLOWERING LOCUS C transcription during thermosensory flowering [112]. Moreover, HOS1 functions as a transcriptional coregulator, restricting the transcriptional activation of PHYTOCHROME INTERACTING FACTOR 4 (PIF4), is a key transcription factor that stimulates hypocotyl growth [113]. In addition, HOS1 acts as an E3 ubiquitin ligase and is involved in degradation of transcription factor ICE1 and CONSTANS to negatively regulate plant cold stress and confer precocious flowering by cooperation with the NPC [114–117]. HOS1 has been reported to act as a genome protector and can be thermostabilized by a heat shock protein 90 (HSP90). Upon heat stress, HOS1 stimulates thermotolerance by activating genes encoding DNA repair components, such as DNA helicase RECQ2 [118]. These findings together point out a wide range of roles for HOS1 in regulating genome activity. The functions of other ORC components in regulating gene expression have been documented in several comprehensive reviews [24,87].

### The plant NPC membrane ring nucleoporins are involved in gene expression regulation

By combining *in situ* and in cellulo cryo–electron tomography (cryo-ET) and powerful artificial intelligence (AI)-based structure predictions, the membrane ring (MR) of the NPC was elucidated. It forms a transmembrane interaction center that anchors the surface of the inner ring and orients toward the outer ring [119] (Figure 1d). The MR composition, featured by transmembrane helixes, is not a relatively conserved NPC subcomplex among eukaryotes.

Constitutive Expresser of Pathogenesis Related Gene 5 (CPR5) is a plant-specific transmembrane nucleoporin that negatively regulates the immune response [120,121]. Upon effector triggered immunity (ETI) signal transduction, CPR5 undergoes conformational switching from an oligomer to a monomer, in order to permeabilize the NPC by affecting the selective barrier. This conformational switch can facilitate nuclear accumulation of both stress and cell cycle-related signaling cargos and simultaneously induce the release of cyclin-dependent kinase inhibitors SIAMESE and SIAMESE-RELATED 1 (SIM/SMR1), which trigger activation of cell cycle transcription factor E2Fs to activate defense gene expression [120,121]. By screening for modifiers of *cpr5* mutant, Xu et al. reported that nuclear transport receptor Exportin-4 (XPO4), is a genetic enhancer of CPR5. XPO4 specifically transports transcriptional corepressors TOPLESS (TPL) and TPL-related (TPR), which regulate transcription by linking transcription factors, with chromatin remodeling complexes, to modulate immune signaling downstream of CPR5 during ETI induction [122]. Intriguingly, CPR5 was added to the list as a novel RNA-binding protein [123]. CPR5 interacted with the RNA splicing activator NineTeen Complex and RNA polyadenylation factor. CPR5 contains an RNA recognition motif, which can affect many alternatively spliced genes (ASGs). This study proposed that CPR5 is a novel component of RNA processing complexes and works in the pre-mRNA splicing machinery in response to the plant immune response [123]. PLANT NUCLEAR ENVELOPE TRANSMEMBRANE 1 (PNET1) is a recently identified MR nucleoporin [56]. Interestingly, PNET1 physically interacted with CPR5 in condensate-like structures at the nuclear periphery. Also, PNET1 displayed genetic interaction with some ORC components, e.g., loss of both PNET1 and HOS1 or NUP160 led to complete sterility in Arabidopsis [56]. It is reasonable to speculate that PNET1 may mediate gene regulation through connections with those nucleoporins. Together, these recent findings highlight that MR nucleoporins are also deeply involved in regulation of gene expression.

## Conclusions and Future Perspectives

A wealth of studies has significantly advanced our vision of the nuclear periphery, as a multi-functional domain. NE constituents engage in diverse cellular processes, especially in genome organization, by directly binding with chromatin and chromatin-associated protein complexes, organizing chromatin architecture at the nuclear periphery, and regulating nuclear accumulation of RNA and proteins. Some converging themes have been elucidated from the structural and functional characterization of NE proteins. The proximity of heterochromatin to NL is one of the most pronounced and conserved features prevailing among eukaryotic genomes. Plant CRWN proteins are responsible for their association with heterochromatin and silenced regions, thus establishing a repressive environment for gene expression. Moreover, CRWN proteins function as a platform for docking diverse chromatin regulators, such as chromatin remodelers, INM proteins, and NB components. However, many gaps in our knowledge remain pertaining to mechanisms that orchestrate the recognition, sequestration, organization, and functionality of peripheral heterochromatin.

Even though many plant NE proteins have been identified, knowledge on the composition of plant INM and their regulatory mechanisms in coordinating genome activity is still largely incomplete, relative to research in animal and yeast cells. The study of PNET2 represents an example in exploring this aspect. We envision that a more extensive investigation of the plant INM composition and its function will shed more light on the importance of plant INM in regulating genome architecture and activity.

As an ancient structural component of eukaryotic cells, much progress on the NPC structure has been implicated in animals, yeast, and algae. Yet, such information is still largely missing in higher plants. Solving the NPC structure of higher plants, particularly of the NB and membrane ring, that contain a myriad of plant-specific NPC components, is a challenging but exciting direction for the future. Furthermore, we currently have little information about the specific roles of different plant nuclear pore components in the regulation of chromatin and gene expression. Deciphering the molecular and functional principles of these plant nucleoporins will help integrate how the NPC governs genome regulation and different signaling pathways in plants.
